# A Switch in the Dynamics of Intra-Platelet VEGF-A from Cancer to the Later Phase of Liver Regeneration after Partial Hepatectomy in Humans

**DOI:** 10.1371/journal.pone.0150446

**Published:** 2016-03-01

**Authors:** Bibek Aryal, Toshiaki Shimizu, Jun Kadono, Akira Furoi, Teruo Komokata, Maki Inoue, Shunichiro Ikeda, Yoshihiko Fukukura, Masatoshi Nakamura, Munekazu Yamakuchi, Teruto Hashiguchi, Yutaka Imoto

**Affiliations:** 1 Cardiovascular and Gastroenterological Surgery, Graduate School of Medical and Dental Sciences, Kagoshima University, Kagoshima, Japan; 2 Department of Laboratory and Vascular Medicine, Graduate School of Medical and Dental Sciences, Kagoshima University, Kagoshima, Japan; 3 Department of Surgery, Kirishima Medical Center, Kirishima, Japan; 4 Department of Surgery, Kagoshima Medical Center, National Hospital Organization, Kagoshima, Japan; 5 Department of Radiology, Kirishima Medical Center, Kirishima, Japan; 6 Department of Radiology, Graduate School of Medical and Dental Sciences, Kagoshima University, Kagoshima, Japan; 7 Department of Clinical Laboratory, Kagoshima University Medical and Dental Hospital, Kagoshima, Japan; University of Bari Medical School, ITALY

## Abstract

**Background:**

Liver regeneration (LR) involves an early inductive phase characterized by the proliferation of hepatocytes, and a delayed angiogenic phase distinguished by the expansion of non-parenchymal compartment. The interest in understanding the mechanism of LR has lately shifted from the proliferation and growth of parenchymal cells to vascular remodeling during LR. Angiogenesis accompanied by LR exerts a pivotal role to accomplish the process. Vascular endothelial growth factor (VEGF) has been elucidated as the most dynamic regulator of angiogenesis. From this perspective, platelet derived/Intra-platelet (IP) VEGF-A should be associated with LR.

**Material and Methods:**

Thirty-seven patients diagnosed with hepatocellular carcinoma and undergoing partial hepatectomy (PH) were enrolled in the study. Serum and IP VEGF-A was monitored preoperatively and at four weeks of PH. Liver volumetry was determined on computer models derived from computed tomography (CT) scan.

**Results:**

Serum and IP VEGF-A was significantly elevated at four weeks of PH. Preoperative IP VEGF-A was higher in patients with advanced cancer and vascular invasion. Postoperative IP VEGF-A was higher after major liver resection. There was a statistically significant correlation between postoperative IP VEGF-A and the future remnant liver volume. Moreover, the soluble vascular endothelial growth factor receptor-1 (sVEGFR1) was distinctly down-regulated suggesting a fine-tuned angiogenesis at the later phase of LR.

**Conclusion:**

IP VEGF-A is overexpressed during later phase of LR suggesting its implications in inducing angiogenesis during LR.

## Introduction

Liver regeneration is a well-orchestrated phenomenon essential for both acute restoration of liver volume after resection and maintenance of its volume during chronic liver injury. A regenerating liver, after partial hepatectomy (PH), requires the formation of a complex network of liver sinusoids through which the blood flows. Thus, the angiogenic phase is a fundamental process of LR, occurring predominantly in the later phase [1]. Among the seven members of the vascular endothelial growth factor (VEGF) family (VEGF-A, VEGF-B, VEGF-C, VEGF-D, VEGF-E, VEGF-F, and PlGF), VEGF-A lies at the forefront of blood vessels formation[2, 3]. VEGF-A is known as the most potent and specific growth factor for both angiogenesis and vasculogenesis[4, 5]. Importantly, VEGF-A holds a regulatory role in differentiation and growth of liver sinusoidal endothelial cells (LSECs) [6]. VEGF-A has been considered as a central angiogenic player of LR[7].

Liver injury elicits trapping and accumulation of platelets in the liver, where they adhere to the endothelium and this interaction is considered to activate LSECs by the release of various growth factors including VEGF-A [8]. At the same time, different growth factors released from activated platelets are also responsible for the proliferative effects on hepatocytes[8]. Notably, recent *in vitro* study suggests that platelets deliver their RNA content to the hepatocyte and this RNA transfer contributes to platelet-mediated hepatocyte proliferation[9]. A new novel mechanism by which platelets communicate with their environment has been known, which involves the *de novo* protein synthesis[10] rather than catching from the circulation.

Platelet secreted VEGF-A, as a robust angiogenic factor, has gained significant attention for its grievous role in tumor biology[11–14]. Platelets also modulate wound healing and tissue regeneration by release of its growth factors like VEGF-A[15]. There should be a discernible variation between the pathological and physiological angiogenesis propelled by the platelet VEGF-A. In this study, we assessed the level of IP VEGF-A preoperatively and at four weeks of PH in patients with hepatocellular carcinoma (HCC). As a result, a significantly higher level of IP VEGF-A was observed at four weeks of PH. Preoperative IP VEGF-A showed positive association with HCC progression. The IP VEGF-A level was noted prominent during the later phase of LR and the elevation was associated with the future remnant liver volume (FRLV).

## Patients and Method

### Prospective Study Cohorts

From May 2013 to December 2014, 37 patients undergoing liver resection were enrolled in the study. To maintain the homogeneity of the cohort, only patients with primary hepatocellular carcinoma (HCC) were included in the study. The diagnosis was made on the basis of abdominal computed tomography (CT), magnetic resonance imaging (MRI), alpha-fetoprotein (AFP) and prothrombin induced by vitamin K absence-II (PIVKA-II). Liver resections were classified according to the International Hepato-Pancreato-Biliary Association, Brisbane (IHPBA) 2000 nomenclature: major hepatectomy defined as resection of 3 or more than 3 Couinauds segments and minor hepatectomy as the resection of less than 3 Couinauds segments.

### Collection of Samples

Venous blood was collected immediately before surgery (PRE OP) and four weeks after surgery (POST OP). Complete blood count (CBC) was performed with an automated hematology analyzer Sysmex XE-5000 (Sysmex Corporation, Kobe, Japan).

The institutional ethics committee (Kagoshima University # 24-155/ 26-77, Kirishima Medical Center # 2505 and Kagoshima Medical Center # 25–30) approved analyses of blood samples and patient data; all patients gave signed, informed consent. All three institutional ethic committee reviewed and specifically approved this study. Study was conducted in accord with the ethical standards of the Committee on Human Experimentation of the institution in which the experiments were done or in accord with the ethical standards of the Helsinki Declaration of 1975.

### Serum and Plasma Preparation

Whole blood was collected in the serum separating tube and a citrate tube, containing 0.5 ml of sodium citrate, and an EDTA-2k tube for cell count (Venoject II, Terumo Corp., Tokyo, Japan). Serum tube was incubated undisturbed at room temperature for 30 minutes to allow clotting. Both serum and plasma tubes were centrifuged at 1710 × g for 10 minutes.

The resultant supernatants were carefully pipetted, at least 5 mm above the clot.

### Platelet Isolation

Blood was drawn in two citrate tubes. The tubes were centrifuged at 90 × g for 15 minutes. The resultant platelet rich plasma (PRP) was gently pipetted with precautions to avoid contamination. Next, the PRP was centrifuged at 2810 × g to isolate the platelets. The supernatant (platelet poor plasma) was collected very precisely and decanted for the complete removal of the plasma from the pellets. Platelet pellets isolated from each 200 μl of PRP were suspended in 220 μl of lysis buffer (150 mM sodium chloride, 25 mM Tris-HCl pH 7.6, 1% Tergitol-type NP-40, 0.1% sodium dodecyl sulfate, 1% sodium deoxycholate); after incubating for 20 minutes, the lysate solution was pipetted and vortexed until the pellets were completely dissolved in the solution.

CBC was carried out in 3 preparations: whole blood, PRP and PPP.

### Quantification of the Growth Factors and Cytokines

Serum, plasma and platelet extracts were analyzed together by commercially available enzyme-linked immunosorbent assay (ELISA) tests for human VEGF-A, HGF, Epidermal growth factor (EGF), Platelet derived growth factor-BB (PDGF-BB), soluble VEGF receptor-1 (sVEGFR-1) and soluble VEGF receptor-2 (sVEGFR-2), Angiopoetin-1 (Ang -1), Interleukin-6 (IL-6) (Quantikine; R&D Systems, Minneapolis, MN, USA) and tumor necrosis factor alpha (TNF-α) (Novex, Life Technologies, Carlsbad, CA, USA); according to the manufacturer’s guidelines.

### Calculation of IP VEGF-A

Platelet content of VEGF-A per 10^6^ platelets was calculated using the equation:

$$=\frac{220\;\mu l\;\times \;VEGF-A\;\left(pg/ml\right)\;in\;platelet\;lysate\;solution}{1000\;\times \;10^{6}platelets/\mu l}\;\;\;\times \frac{1}{(200\;\mu l\;PRP\;\times \;platelet\;count\;in\;PRP\;\left(10^{6}\;platelets/\;\mu l\right)}$$

Intraplatelet (IP) Ang-1 was calculated using the similar equation.

### Liver Function Assessments

Serum bilirubin, albumin, aspartate aminotransferase (AST), alanine aminotransferase (ALT), alkaline phosphatase (ALP), gamma-glutamyl transferase (GGT), alpha-fetoprotein (AFP), C-reactive protein (CRP) and prothrombin time (PT) were measured as routine blood test.

Hepatic functional reserve was assessed by indocyanine green (ICG) clearance test, 99mTc-galactosyl human serum albumin (GSA) scintigraphy and Child-Pugh score.

Preoperative liver volumetric analyses were obtained using a three-dimensional volume analyzer (SYNAPSE VINCENT; FUJIFILM Medical Co., Tokyo, Japan). Resection volume or FRLV was expressed in percentage. Out of 37 patients, 33 patients underwent liver volumetric examination and were used in the analysis with postoperatively detectable variables.

### Statistical Analysis

Statistical analyses were conducted using Graph Pad Prism (version 6.0d for Mac OS X, USA, GraphPad software, San Diego California, USA) and were based on nonparametric tests (Mann-Whitney’s U test, Wilcoxon’s test, and Spearman’s correlation). IP VEGF-A and IP Ang-1 level were expressed per 10^6^ platelets; all the biomarkers are shown graphically using box-and-whisker plots. Two-tailed *P* values of less than 0.05 were considered statistically significant.

## Results

### Patient Demographics

Clinicopathological characteristics of all patients are summarized in Table 1. Although the cohort included only patients with HCC, the evidence of cirrhosis was not evenly distributed. All the patients in the study belonged to Child Pugh class A according to the Child-Pugh classification. Nineteen patients received preoperative transcatheter arterial chemoembolization (TACE) or radiofrequency ablation (RFA). None of the patients received intraoperative platelet transfusions. Taking preoperative IP VEGF-A as a reference, 10 baseline characters (age, sex, etiology, cirrhosis, tumor staging, tumor size, vascular invasion, previous interventions, resection type and serum AFP) were analyzed. Except for tumor size, staging, vascular invasion and resection group, no statistically significant difference in baseline characteristics was observed between the groups (Table 1)

**Table 1:** Clinicopathological characteristic of patients with HCC with reference to preoperative IP VEGF-A.

Parameter	N = 37	P-Value
**Age**		0.3093
≥65	28	
<65	9	
**Sex**		0.2137
Male	28	
Female	9	
**Etiology**		0.551
HBV +	11	
HCV +	12	
Non-viral	14	
**Cirrhosis**		0.613
+	20	
−	17	
**Tumor staging**		0.0043
(I/II)	27	
(III/IV)	10	
**Tumor size**		0.0095
<5 cm	26	
≥5 cm	11	
**Vascular invasion**		0.0044
No	30	
Yes	7	
**Previous Treatment**		0.95
TACE/RFA	19	
NONE	18	
**Resection Type**		0.0027
Minor	25	
Major	12	
**Serum αFP(ng/ml)**		0.7489
<50 ng/ml	25	
≥50 ng/ml	12	

### IP VEGF-A is Elevated in the Later Phase of LR

Similarly to previous findings, serum contained the major pool of circulating VEGF-A (*P* = 0.001; r = 0.378; S1A Fig), and the serum VEGF-A was directly correlated with the platelet count (*P*<0.01; r = 0.302; S1B Fig). There was a marginally significant difference between the preoperative and postoperative platelet counts (median platelet count: PRE OP, 14.80 ×10^4^ platelets; POST OP, 16.30 ×10^4^ platelets; *P* = 0.08; S2A Fig), however, no significant difference in the platelet counts was observed between the subset of major and minor hepatectomy group (median platelet count: PRE OP major, 15 ×10^4^ platelets; PRE OP minor, 14.4 ×10^4^ platelets; *P* = 0.66 and median platelet count: POST OP major, 15.10 ×10^4^ platelets; POST OP minor, 17.3 ×10^4^ platelets; *P* = 0.7; S2B Fig).

**Fig 1:**
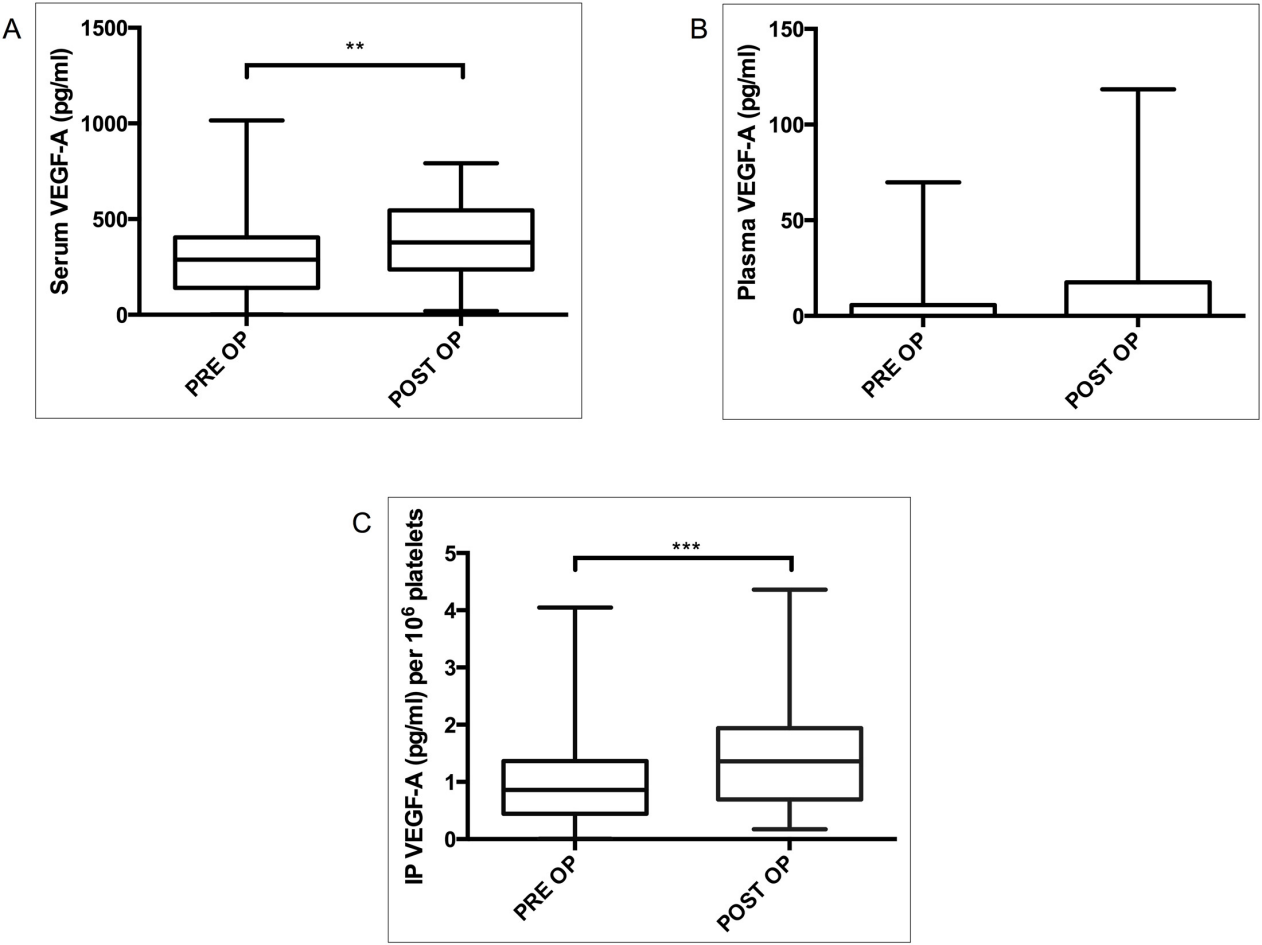
VEGF-A before and after surgery in serum (A), plasma (B) and IP (C). Samples were collected before (PRE OP) and four weeks after partial hepatectomy (POST OP). IP VEGF-A concentration was expressed per 10^6^ platelets. *P≤0.*05*; **P≤ 0.01; ***P≤0.001.

The study focused on the angiogenic or delayed phase of LR. Serum VEGF-A was analyzed in two events: preoperatively and at four weeks after PH; the postoperative level of serum VEGF-A was higher than that of the preoperative level (median serum VEGF-A: PRE OP, 288.4 pg/ ml; POST OP, 377.7 pg/ml, *P*<0.01; Fig 1A). Similarly, the postoperative IP VEGF-A was significantly more elevated than that of the preoperative level (median IP VEGF-A × 10^6^ platelets: PRE OP, 0.8612 pg; POST OP, 1.362 pg, *P*<0.001; Fig 1C). There was no significant difference in the level of plasma VEGF-A between preoperative and postoperative states (*P* = 0.495; Fig 1B). This findings show that both serum and IP VEGF-A is elevated at four weeks after PH.

### Association of IP VEGF-A with Tumor Size and RLV

**Fig 2:**
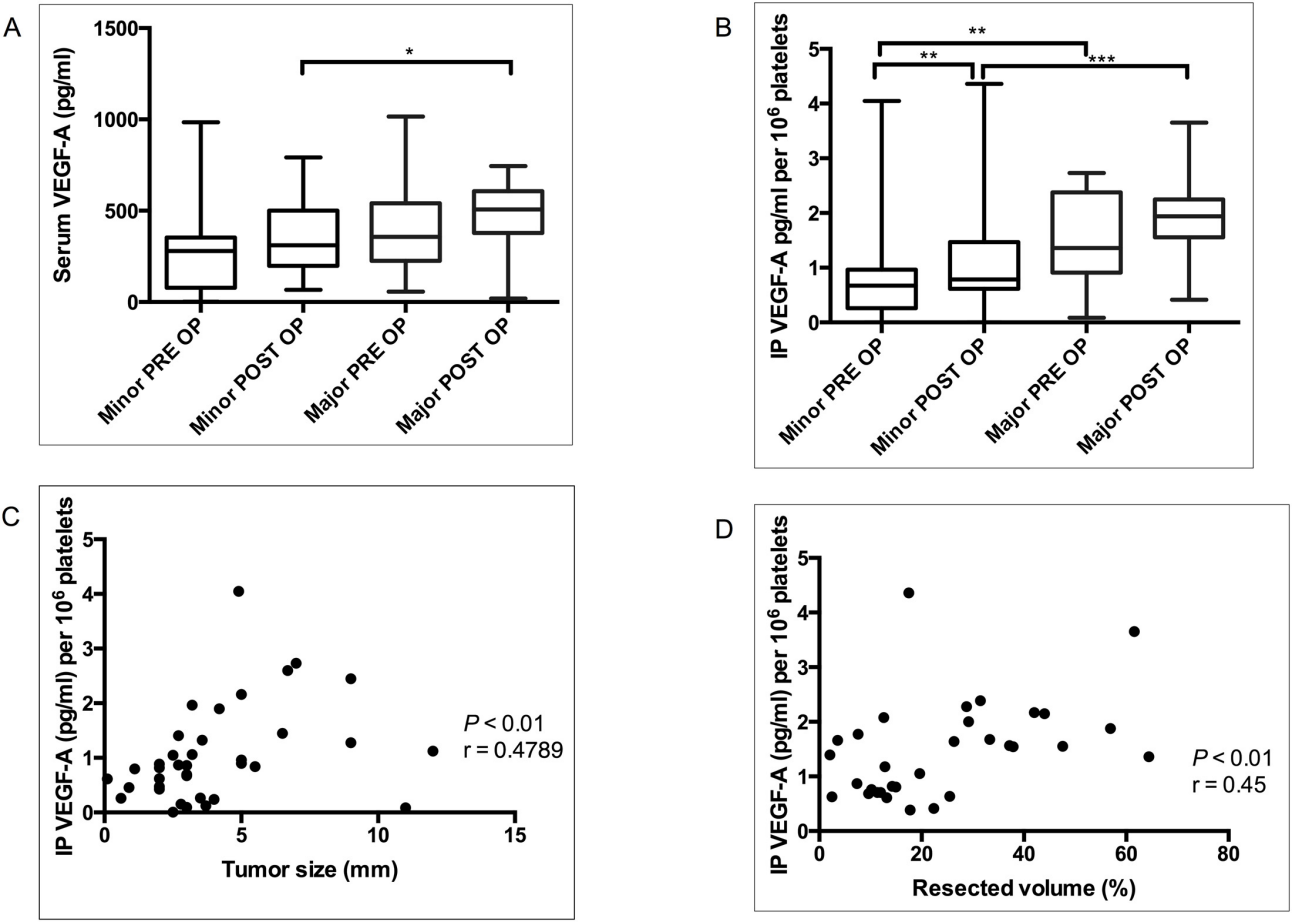
Difference in of serum (A) and IP VEGF-A (B) in major and minor hepatectomy groups. The levels were compared before (PRE OP) and after four weeks of partial hepatectomy (POST OP). Scatter plot showing correlation between: preoperative IP VEGF-A and tumor size (C), postoperative IP VEGF-A and resection volume (D) (expressed in percentage). IP VEGF-A concentration was expressed per 10^6^ platelets. *P≤0.*05*; **P≤ 0.01; ***P≤0.001.

Patients were divided in two groups; minor and major hepatectomy groups. The difference in concentrations of serum and IP VEGF-A were compared in the two groups (Fig 2A and 2B). Even though the preoperative level of serum VEGF-A in the major group was apparently higher than that of the minor group, no statistically significant difference was observed between the two groups (median serum VEGF-A: PRE OP major, 398.0 pg/ml; PRE OP minor, 275.4 pg/ml; *P* = 0.082), but the postoperative serum VEGF-A in the major group was significantly higher than that of the minor group (median serum VEGF-A: POST OP major, 485.0 pg/ml; POST OP minor, 345.6 pg/ml; *P*<0.05).

IP VEGF-A followed a similar trend like serum VEGF-A; as shown in Fig 2B, preoperative IP VEGF-A level in the major group was significantly higher than that of the minor (median IP VEGF-A × 10^6^ platelets: PRE OP major, 1.362 pg; PRE OP minor, 0.619 pg; *P*<0.01). The postoperative IP VEGF-A in the major group was significantly elevated as compared to the minor group (median IP VEGF-A × 10^6^ platelets: POST OP major, 1.938 pg; POST OP minor, 0.786 pg; *P*<0.001). In the subset of minor group, IP VEGF-A was significantly elevated postoperatively (median IP VEGF-A × 10^6^ platelets: PRE OP minor, 0.619; POST OP minor, 0.786: *P*<0.01). Despite the fact that 75% of the cases had postoperatively elevated IP-VEGF in the major group, statistically we observed only a marginal significant difference between the preoperative and postoperative IP-VEGF A in the major subset (median IP VEGF-A × 10^6^ platelets: PRE OP major, 1.362, pg/ml; POST OP major, 1.938 pg/ml: *P* = 0.09).

Further, we specifically analyzed the correlation between tumor size and preoperative IP VEGF-A. The relationship between resection volume percentage and postoperative IP VEGF-A was analyzed to check the impact of RLV on IP VEGF-A sequestration. We found a significant correlation between the preoperative IP VEGF-A and the tumor size (*P*<0.01; r = 0.4789; Fig 2C); similarly postoperative IP VEGF-A and resection volume also showed a statistically significant correlation (*P*<0.01; r = 0.452; Fig 2D). These findings demonstrate that the volume of IP VEGF-A is associated with tumor size and resected volume.

### Association of Pro-inflammatory Cytokine IL-6 in IP VEGF-A Sequestration

**Fig 3:**
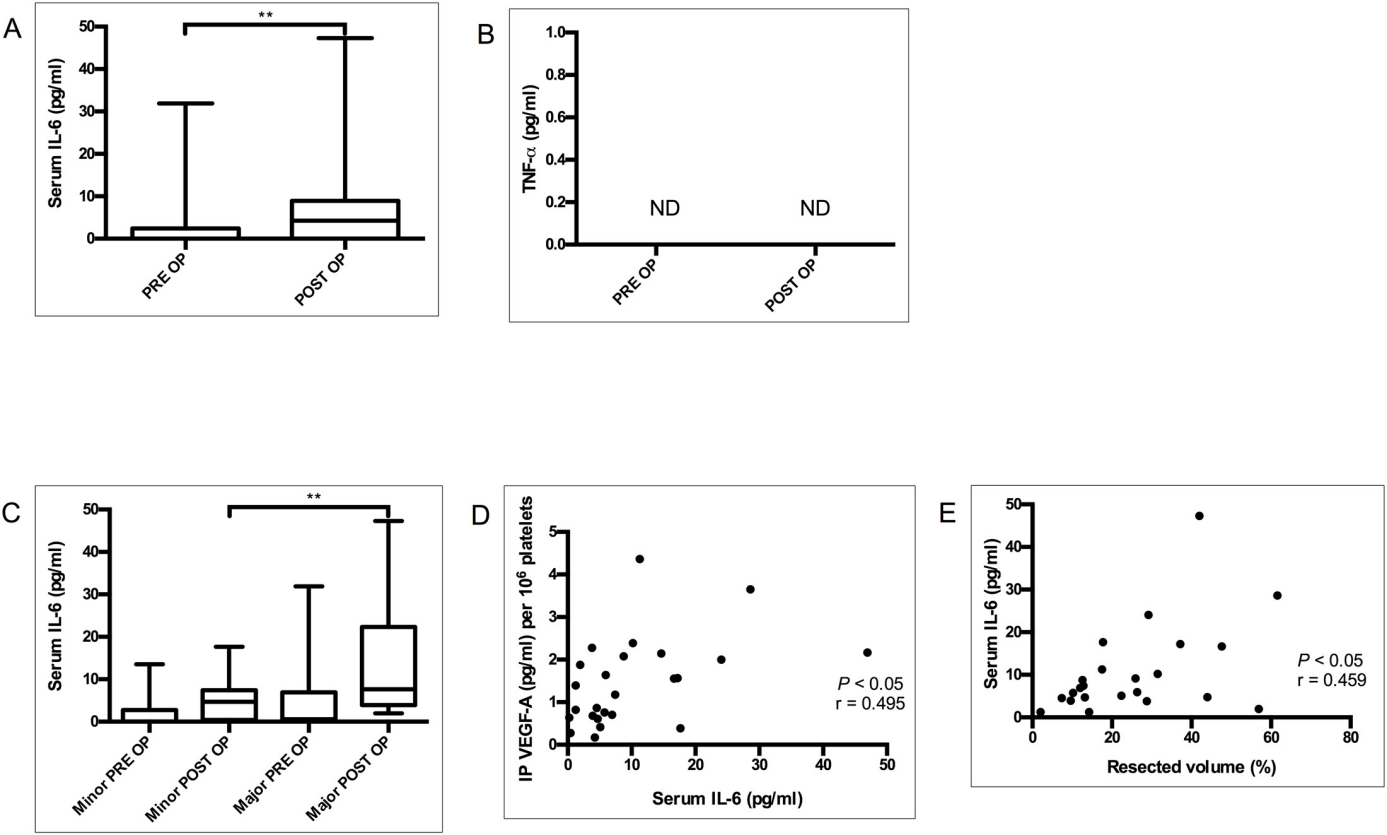
Pro-inflammatory markers of LR. Serum concentrations of IL-6 (A) and TNF-α (B) were analyzed preoperatively (PRE OP) and four weeks after partial hepatectomy (POST OP). Serum IL-6 in major and minor hepatectomy group (C) (N = 25). Correlation between postoperatively elevated serum IL-6 and IP VEGF-A (D). Correlation between postoperative IP VEGF-A and resection volume (E) (expressed in percentage). IP VEGF-A concentration was expressed per 10^6^ platelets (N = 25). *P≤0.*05*; **P≤ 0.01; ***P≤0.001. ND = Not detected.

Previous studies have shown the role of IL-6 in VEGF-A production[16, 17]. Of 37 cases, IL-6 was detectable in postoperative serum in 25 patients. The postoperative serum IL-6 level was significantly elevated compared to the preoperative level (median serum IL-6: PRE OP, 0 pg/ml; POST OP, 4.246 pg/ml; *P*< 0.01; Fig 3A). However, serum TNF- α level was below the detectable range in both preoperative and postoperative samples (Fig 3B).

Compared to the minor hepatectomy group, the major hepatectomy group demonstrated a more pronounced elevation of serum IL-6 (median serum IL-6: Major, 10.18 pg/ml; minor, 4.70 pg/ml; *P*<0.05) during LR as illustrated in Fig 3C. Elevation of serum IL-6 is obvious for several days following major surgery, however elevation of IL-6 at one month of hepatectomy was not predicted. We found a statistically significant correlation between postoperative IL-6 and IP VEGF-A (*P*<0.05; *r* = 0.4905; Fig 3D). Additionally, the significant correlation between postoperative serum IL-6 and the percentage of volume resection (*P*<0.05; *r* = 0.459; Fig 3E) corresponds to the similar association of IP VEGF-A and the extent of resection. Taken together, IL-6 is found to be elevated after four weeks of PH, and correlates with IP VEGF-A.

### Major Mitogens and Other Key Platetet Derived Growth Factors are not Elevated in the Later Phase of LR

**Fig 4:**
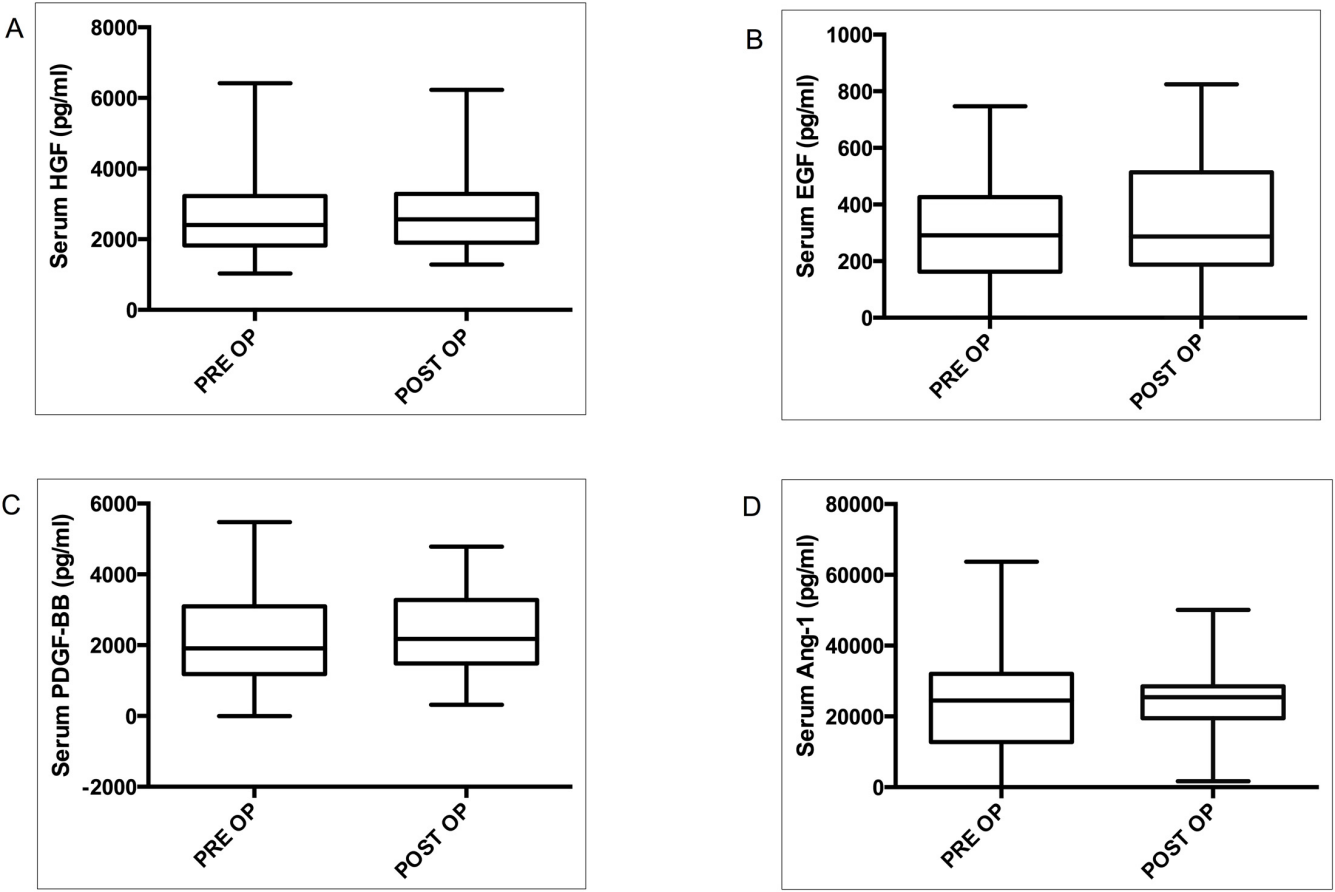
Major Mitogens and other central platetet derived growth factors are not elevated in the later phase of LR. Serum concentration of major mitogens, HGF (A) and EGF (B) were analyzed before surgery (PRE OP) and four weeks after partial hepatectomy (POST OP). Serum concentration of PDGF-BB (C) and Ang-1 (D) were analyzed before surgery (PRE OP) and 4 weeks after surgery (POST OP).

Hepatocyte growth factor (HGF) and epidermal growth factors (EGF) are the major mitogens; the breach in the signaling pathways associated with these factors would likely abolish the process of LR[18]. No statistically significant change in serum HGF concentrations between the preoperative and postoperative phases was found (median serum HGF: PRE OP, 2401 pg/ml; POST OP = 2565 pg/ml; *P* = 0.511; Fig 4A). Serum EFG is largely secreted by platelets. We also found a significant positive correlation between platelet count and serum EGF (S3A Fig). Serum EGF also did not yield a significant difference between the two phases (median serum EGF: PRE OP = 291.3 pg/ml; POST OP = 287.5 pg/ml; *P* = 0.40; Fig 4B). Platelet derived growth factor (PDGF)-BB, known as the most potent mitogen for hepatic stellate cell proliferation, is substantially produced by platelets. Not surprisingly, serum PDGF-BB was positively correlated with the platelet count (*P*<0.001; r = 0.41; S3B Fig). We didn't find a significant difference in the serum concentration of PDGF between preoperative and postoperative states (median serum PDGF-BB: PRE OP, 1912 pg/ml; POST OP, 2178 pg/ml; *P* = 0.30; Fig 4C).

Next, we assessed angiopoetin (Ang)-1 as another important marker of angiogenesis during LR[19]. A major portion of serum Ang-1 derives from the platelet[20]. A direct correlation between serum Ang-1 and platelet count was found (*P*<0.01; r = 0.32;S3C Fig). Surprisingly, no significant difference was observed in the level of serum Ang-1 between preoperative and postoperative phases (median serum Ang1: PRE OP = 24504; POSTOP = 25449 pg/ml; *P* = 0.79; Fig 4D). Additionally, analysis of IP Ang-1 from the isolated platelets also did not demonstrate any significant difference between preoperative and postoperative concentrations (median IP Ang1 × 10^6^ platelets: PRE OP = 13688 pg/ml; POST OP = 15262 pg/ml; *P* = 0.676; S3D Fig). Collectively, these findings suggest that there is a selective elevation of VEGF-A in the later phase of LR.

### Depletion in the Level of Soluble VEGF Receptor-1 in the later Phase of LR Inversely Correlates With The Resection Volume

**Fig 5:**
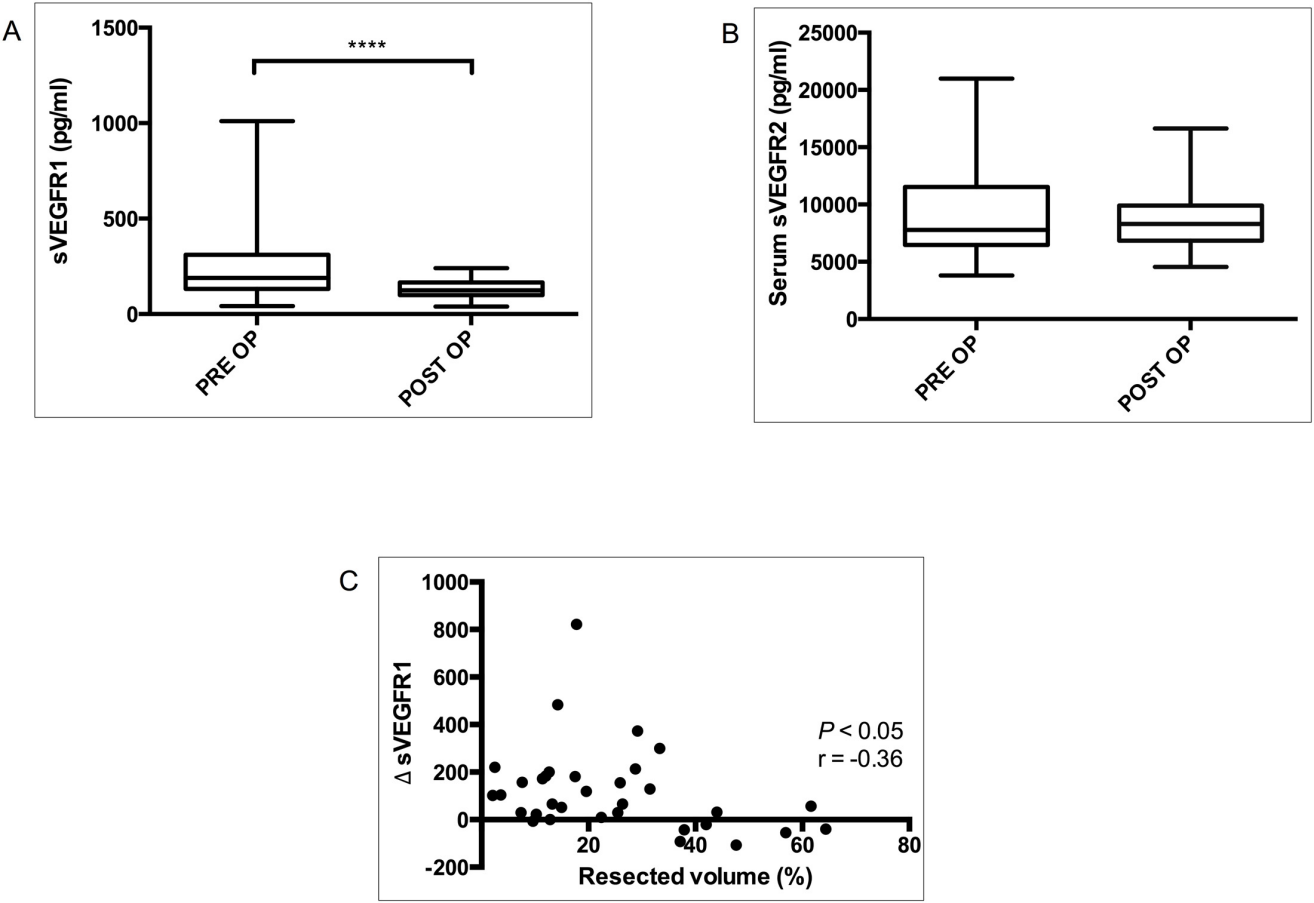
Soluble VEGF receptors during angiogenic phase of LR. Soluble VEGF receptor-1 (A) and soluble VEGF receptor-2 (B) evaluated before surgery (PRE OP) and four weeks after partial hepatectomy (POST OP). Correlation between delta sVEGFR-1 and resection volume (C). *P≤0.*05*; **P≤ 0.01; ***P≤0.001, ****P< 0.0001.

Soluble VEGF receptors determine the level of circulating VEGF-A [21]. Our results portray an ongoing angiogenic drive at one month of hepatectomy. We observed a significant drop in the level of postoperative sVEGFR1 than that of the preoperative (median sVEGFR1: PRE OP, 189.6 pg/ml; POST OP, 124.3 pg/ml; *P*< 0.0001; Fig 5A). Unlike sVEGFR1, we did not observe significant difference in the level of serum sVEGFR2 between the two events (median sVEGFR2: PRE OP, 7773 pg/ml; POST OP, 8306 pg/ml; *P* = 0.976; Fig 5B). No direct association between soluble VEGF receptors and IP VEGF-A was noted. Strikingly, however, the sharp drop in the level of sVEGFR1 (delta sVEGFR1) was inversely correlated with the resection volume (*P<0*.*05; r* = -0.3620; Fig 5C).

## Discussion

Two distinct phases of LR after PH have been reported: an early inductive phase with rapid proliferation of hepatocytes followed by an angiogenic phase distinguished by the expansion of non-parenchymal compartment. Our study examined IP VEGF-A in the later phase of LR.

Major serum pool of VEGF-A is attributed to the activated platelets in cancers and other pathologies[12, 13, 22]. Serum VEGF-A per platelet count has been studied in HCC patients, illustrating its association with disease progression [13]. Our study analyzed the actual VEGF-A content in isolated platelets from patients with HCC and observed its association with cancer progression. There is ample evidence that platelets are directly involved in tumor growth, angiogenesis and metastasis. The adverse effects of platelet on HCC are essentially due to the growth factors secreted from its granules[23]. Furthermore, another study has suggested that VEGF may improve LR after PH [8]. Notably, we have demonstrated prominent elevation of serum and IP VEGF-A at a remote phase of LR. Our findings forward an insight on dual roles of IP VEGF-A on HCC and LR.

Surgical wound induces the initiation of local and systemic reactions, where activated platelets secrete various growth factors to repair the injured structures[15]. Analysis in the early phase after major surgery would not solely reflect platelets response to LR, as surgical wound healing is active for the first few weeks of operation. Bondestam J et al.[24] observed elevation of serum VEGF-A after major surgery on the first and third postoperative day, but not in the later samples. In the current study, the cytokines were analyzed four weeks after surgery, when the CRP level had already reverted to normal (S4 Fig), minimizing the chances of overlap between platelets response to surgical wound healing and LR. An active regeneration with increased mitotic activity has been demonstrated until 10–35 days following hepatectomy in normal livers, whereas the livers with cirrhosis or hepatitis showed histologic evidence of regeneration during the first two months [25]. Although the major volume regenerates by one month, LR continues throughout the first postoperative year[26]. Most postoperative liver failures are known to occur within a month of liver resection[27]. Four weeks after PH is a decisive period of LR. Furthermore, the aim of this study was to examine the IP VEGF-A at the later phase of LR when angiogenesis is supposed to be more prominent.

The circulating levels of the major mitogens of hepatocytes proliferation, HGF and EGF[18], did not show significant changes in the serum at four weeks of PH. Other major angiogenic factors, EGF, Ang-1 and PDGF-BB, considered essential in LR[19], are largely secreted by platelets[20]. PDGF-BB is known for its potent proliferative effect on hepatic stellate cells[28]. Nevertheless, lack of significant changes in the level of major growth factors including those derived from the platelet itself reflect perpetual potential of IP VEGF-A in modulating angiogenesis during the later phase of LR.

The two major pro-inflammatory cytokines IL-6 and TNF-α are centrally responsible for activation of STAT-3, NF-κB, phosphoinositide 3-kinase (PI3K) and Akt pathways during LR[29, 30]. Deficiency of one of these cytokines in the bone marrow derived blood cells significantly impaired LR in mice[31]. The relationship between IL-6 and VEGF-A production has already been established in different cell models[16, 17, 32]. That expression of VEGF-A is regulated by IL-6 in human megakaryoblastic cell line MEG-01[33] prompts speculation that IP VEGF-A may derive from the megakaryocytes. Except for its proliferative competency in the early phase of LR, IL-6 might enhance angiogenesis during the later phase of LR by promoting IP VEGF-A production.

Circulating levels of sVEGFR1 essentially determine the pathophysiological roles of VEGF-A as it binds to free VEGF-A, reducing the actual amount binding to endothelium[34]. Furthermore, sVEGFR1 as an antiangiogeneic agent significantly reduces the number of LSECs after PH, thereby suppressing LR[35]. Angiogenesis relies on a balance between positive and negative endothelial regulators [36]. The suppression of sVEGFR1 observed in our study reflects a fine-tuned angiogenesis during the later phase of LR.

Clinical evidence of platelets as relevant inducer of LR has been demonstrated in human subjects[37, 38]. Intrahepatic accumulation of platelets was observed in mice models[39]; in a more recent study, Starlinger et. al [38] have reported that platelets selectively accumulate at the site of LR after PH in human subjects. Although the underlying mechanism of intrahepatic accumulation is not completely understood, it is presumed that a potential trigger for platelet migration and trapping within the liver could be the hemodynamic alterations generated in the remnant liver by the surgical procedure. The platelet activation might be elicited by the shear stress itself or by platelet binding to an as yet unidentified membrane-bound receptor on LSEC or hepatocytes, or by platelet internalization[8]. Regardless of the solid underlying mechanism, it is now acknowledged that the intrahepatic accumulation of platelet following injury has a mitogenic effect on LR. Platelets proliferative effect on liver cells is attributed to release of its growth factor and transfer of platelets RNA[9].

In our study, even though most of the patients had an elevated postoperative platelet count, there was no statistically significant difference between the counts of pre and postoperative events nor was any obvious difference observed between the platelet count of major and minor group (S2 Fig). Depending on the RLV, however, each platelet was loaded with an excess amount of VEGF-A. The IP VEGF-A, described as excess in cancers[11–14], is demonstrated here to be much higher after a month of PH.

In a large cohort of patients with gastric cancer, radical resection of the tumor resulted in a significant decrease in preoperative serum VEGF-A over a 30-day period [40]. In another study, serum VEGF-A raised for the first 3–5 days, but not in later days after major cardiovascular surgery in patients without cancer[24]. On the contrary, we found substantial elevation in the concentration of VEGF-A in a month of PH, indicating its relevance to the later phase of LR. As a limitation of our study, the degree of neo-angiogenesis was not assessed in the resected specimen or regenerating liver. However, prominent elevation in IP VEGF-A concentration coupled with significant down-regulation of neutralizing sVEGFR1 maintained a sustained pro-angiogenic environment for neovascularization during the later phase of LR.

VEGF-A, up-regulated in various cancers, is closely correlated with pathological characteristics of the tumor, metastasis and prognosis. Our findings showed association of IP VEGF-A with HCC tumor size, stage and vascular invasion. Taken together, these results suggests that preoperative IP VEGF-A might represent a potential predictor of HCC progression. On the other hand, angiogenesis accompanied by LR is indispensible for the sustained regeneration. Multiple angiogenic factors are required following liver resection or liver injury, and VEGF-A as a pro-proliferative mitogen is essential during LR. Moreover, a recent study has indicated an important association between defective releases of platelet VEGF-A and liver dysfunction after PH in humans[38]. VEGF-A dominantly behaves as a complete mitogen for LSECs[41]. We found selective elevation VEGF-A in the delayed phase of LR, while other major pro-proliferative mitogens like HGF, EGF, Ang-1 and PDGF-BB were not significantly changed to the preoperative level. These findings suggest that competency of IP VEGF-A in facilitating the angiogenesis until later phase of LR. Administration of VEGF-A increased liver mass in mice model[42]. A conceivable implication of exogenous VEGF-A has also been discussed in liver disease and in conditions with diminished hepatic signaling[43]. Recruiting IP VEGF-A as an alternative source might lead to a more amenable impact on LR. The paradoxical response of IP-VEGF-A to HCC and the later phase of LR illustrated in this study, takes one step closer to understanding the therapeutic strategies platelet can offer as an inducer of LR.

## Supporting Information

S1 Fig Serum VEGF-A is derived from platelets. Correlation between serum and plasma VEGF-A (A). Correlation between platelet count and serum VEGF-A (B). (Variables included from both preoperative and postoperative events).
(TIFF)

S2 Fig No significant differences in baseline platelet counts between major and minor hepatectomy groups. Platelet count before (PRE OP) and four weeks after operation (POST OP) (A). Platelet count in major and minor groups, before and four weeks after operation (B).
(TIFF)

S3 Fig Profile of platelet derived growth factors. Correlation between platelet count and serum EGF (A), PDGF-BB (B), Ang-1 (C). IP Ang-1 concentrations were analyzed preoperatively (PRE OP) and postoperatively (POST OP) (D). IP Ang-1 was expressed per 10^6^ platelets.
(TIFF)

S4 Fig No statistical difference in the level of CRP between preop and postop group. CRP level before (PRE OP) and four weeks after operation (POST OP) (N = 28).
(TIFF)

## Abbreviations

HCC: hepatocellular carcinoma
LR: liver regeneration
PH: partial hepatectomy
VEGF-A: vascular endothelial growth factor-A
TACE: transcatheter arterial chemoembolization
RFA: radiofrequency ablation
IP: intraplatelet
ALT: alanine aminotransferase
ALP: alkaline phosphatase
AST: aspartate aminotransferase
GGT: gamma-glutamyl transferase
CRP: C-reactive protein
HGF: hepatocyte growth factor
EGF: epidermal growth factor
sVEGFR: soluble VEGF receptor
Ang-1: angiopoetin-1
IL-6: interlukin-6
TNF-α: tissue necrotic factor alpha
PDGF-BB: platelet derived growth factor-BB
ELISA: enzyme-linked immunosorbent assay
ICG: indocyanine green
PRE OP: preoperative
POST OP: postoperative
RLV: remnant liver volume
FRLV: future remnant liver volume

## References

[1] Hu J, Srivastava K, Wieland M, Runge A, Mogler C, Besemfelder E, et al Endothelial cell-derived angiopoietin-2 controls liver regeneration as a spatiotemporal rheostat. Science. 2014;343(6169):416–9. 10.1126/science.1244880 . 24458641

[2] Ferrara N, Gerber HP, LeCouter J. The biology of VEGF and its receptors. Nature medicine. 2003;9(6):669–76. 10.1038/nm0603-669 . 12778165

[3] Leung DW, Cachianes G, Kuang WJ, Goeddel DV, Ferrara N. Vascular endothelial growth factor is a secreted angiogenic mitogen. Science. 1989;246(4935):1306–9. . 2479986 10.1126/science.2479986

[4] Mustonen T, Alitalo K. Endothelial receptor tyrosine kinases involved in angiogenesis. The Journal of cell biology. 1995;129(4):895–8. 7538139 10.1083/jcb.129.4.895 PMC2120485

[5] Shibuya M. Role of VEGF-flt receptor system in normal and tumor angiogenesis. Advances in cancer research. 1995;67:281–316. . 8571818 10.1016/s0065-230x(08)60716-2

[6] Sato T, El-Assal ON, Ono T, Yamanoi A, Dhar DK, Nagasue N. Sinusoidal endothelial cell proliferation and expression of angiopoietin/Tie family in regenerating rat liver. Journal of hepatology. 2001;34(5):690–8. . 11434615 10.1016/s0168-8278(00)00109-4

[7] Bockhorn M, Goralski M, Prokofiev D, Dammann P, Grunewald P, Trippler M, et al VEGF is important for early liver regeneration after partial hepatectomy. The Journal of surgical research. 2007;138(2):291–9. 10.1016/j.jss.2006.07.027 . 17275844

[8] Meyer J, Lejmi E, Fontana P, Morel P, Gonelle-Gispert C, Buhler L. A focus on the role of platelets in liver regeneration: do platelet-endothelial cell interactions initiate the regenerative process? Journal of hepatology. 2015 10.1016/j.jhep.2015.07.002 . 26169159

[9] Kirschbaum M, Karimian G, Adelmeijer J, Giepmans BN, Porte RJ, Lisman T. Horizontal RNA transfer mediates platelet-induced hepatocyte proliferation. Blood. 2015;126(6):798–806. 10.1182/blood-2014-09-600312 . 26056167

[10] Panes O, Matus V, Saez CG, Quiroga T, Pereira J, Mezzano D. Human platelets synthesize and express functional tissue factor. Blood. 2007;109(12):5242–50. 10.1182/blood-2006-06-030619 . 17347408

[11] Verheul HM, Hoekman K, Luykx-de Bakker S, Eekman CA, Folman CC, Broxterman HJ, et al Platelet: transporter of vascular endothelial growth factor. Clinical cancer research: an official journal of the American Association for Cancer Research. 1997;3(12 Pt 1):2187–90. . 9815613

[12] Poon RT, Lau CP, Cheung ST, Yu WC, Fan ST. Quantitative correlation of serum levels and tumor expression of vascular endothelial growth factor in patients with hepatocellular carcinoma. Cancer research. 2003;63(12):3121–6. . 12810638

[13] Kim SJ, Choi IK, Park KH, Yoon SY, Oh SC, Seo JH, et al Serum vascular endothelial growth factor per platelet count in hepatocellular carcinoma: correlations with clinical parameters and survival. Japanese journal of clinical oncology. 2004;34(4):184–90. . 15121753 10.1093/jjco/hyh039

[14] Peterson JE, Zurakowski D, Italiano JE Jr, Michel LV, Connors S, Oenick M, et al VEGF, PF4 and PDGF are elevated in platelets of colorectal cancer patients. Angiogenesis. 2012;15(2):265–73. 10.1007/s10456-012-9259-z . 22402885

[15] Wu FP, Hoekman K, Meijer S, Cuesta MA. VEGF and endostatin levels in wound fluid and plasma after breast surgery. Angiogenesis. 2003;6(4):255–7. 10.1023/B:AGEN.0000029410.32264.b0 . 15166493

[16] Huang SP, Wu MS, Shun CT, Wang HP, Lin MT, Kuo ML, et al Interleukin-6 increases vascular endothelial growth factor and angiogenesis in gastric carcinoma. Journal of biomedical science. 2004;11(4):517–27. 10.1159/000077902 . 15153787

[17] Cohen T, Nahari D, Cerem LW, Neufeld G, Levi BZ. Interleukin 6 induces the expression of vascular endothelial growth factor. The Journal of biological chemistry. 1996;271(2):736–41. . 8557680 10.1074/jbc.271.2.736

[18] Michalopoulos GK. Liver regeneration after partial hepatectomy: critical analysis of mechanistic dilemmas. The American journal of pathology. 2010;176(1):2–13. 10.2353/ajpath.2010.090675 20019184 PMC2797862

[19] Kraizer Y, Mawasi N, Seagal J, Paizi M, Assy N, Spira G. Vascular endothelial growth factor and angiopoietin in liver regeneration. Biochemical and biophysical research communications. 2001;287(1):209–15. 10.1006/bbrc.2001.5548 . 11549276

[20] Brouwers J, Noviyanti R, Fijnheer R, de Groot PG, Trianty L, Mudaliana S, et al Platelet activation determines angiopoietin-1 and VEGF levels in malaria: implications for their use as biomarkers. PloS one. 2014;8(6):e64850 10.1371/journal.pone.0064850 23755151 PMC3670845

[21] Kendall RL, Wang G, Thomas KA. Identification of a natural soluble form of the vascular endothelial growth factor receptor, FLT-1, and its heterodimerization with KDR. Biochemical and biophysical research communications. 1996;226(2):324–8. 10.1006/bbrc.1996.1355 . 8806634

[22] Hashiguchi T, Arimura K, Matsumuro K, Otsuka R, Watanabe O, Jonosono M, et al Highly concentrated vascular endothelial growth factor in platelets in Crow-Fukase syndrome. Muscle & nerve. 2000;23(7):1051–6. . 10882999 10.1002/1097-4598(200007)23:7<1051::aid-mus7>3.0.co;2-v

[23] Goubran HA, Stakiw J, Radosevic M, Burnouf T. Platelet-cancer interactions. Seminars in thrombosis and hemostasis. 2014;40(3):296–305. 10.1055/s-0034-1370767 . 24590421

[24] Bondestam J, Salven P, Jaaskela-Saari H, Ikonen T, Lepantalo M, Mattila S, et al Major surgery increases serum levels of vascular endothelial growth factor only temporarily. American journal of surgery. 2000;179(1):57–9. . 10737580 10.1016/s0002-9610(99)00253-6

[25] Nagasue N, Yukaya H, Ogawa Y, Kohno H, Nakamura T. Human liver regeneration after major hepatic resection. A study of normal liver and livers with chronic hepatitis and cirrhosis. Annals of surgery. 1987;206(1):30–9. 3038039 10.1097/00000658-198707000-00005 PMC1492934

[26] Pomfret EA, Pomposelli JJ, Gordon FD, Erbay N, Lyn Price L, Lewis WD, et al Liver regeneration and surgical outcome in donors of right-lobe liver grafts. Transplantation. 2003;76(1):5–10. . 12865779 10.1097/01.TP.0000079064.08263.8E

[27] Shirabe K, Shimada M, Gion T, Hasegawa H, Takenaka K, Utsunomiya T, et al Postoperative liver failure after major hepatic resection for hepatocellular carcinoma in the modern era with special reference to remnant liver volume. Journal of the American College of Surgeons. 1999;188(3):304–9. . 10065820 10.1016/s1072-7515(98)00301-9

[28] Adachi T, Togashi H, Suzuki A, Kasai S, Ito J, Sugahara K, et al NAD(P)H oxidase plays a crucial role in PDGF-induced proliferation of hepatic stellate cells. Hepatology. 2005;41(6):1272–81. 10.1002/hep.20719 . 15915457

[29] Streetz KL, Luedde T, Manns MP, Trautwein C. Interleukin 6 and liver regeneration. Gut. 2000;47(2):309–12. 10896929 10.1136/gut.47.2.309 PMC1727989

[30] Yamada Y, Kirillova I, Peschon JJ, Fausto N. Initiation of liver growth by tumor necrosis factor: deficient liver regeneration in mice lacking type I tumor necrosis factor receptor. Proceedings of the National Academy of Sciences of the United States of America. 1997;94(4):1441–6. 9037072 10.1073/pnas.94.4.1441 PMC19810

[31] Sudo K, Yamada Y, Saito K, Shimizu S, Ohashi H, Kato T, et al TNF-alpha and IL-6 signals from the bone marrow derived cells are necessary for normal murine liver regeneration. Biochimica et biophysica acta. 2008;1782(11):671–9. 10.1016/j.bbadis.2008.09.010 . 18948191

[32] Dankbar B, Padro T, Leo R, Feldmann B, Kropff M, Mesters RM, et al Vascular endothelial growth factor and interleukin-6 in paracrine tumor-stromal cell interactions in multiple myeloma. Blood. 2000;95(8):2630–6. . 10753844

[33] Salgado R, Benoy I, Weytjens R, Van Bockstaele D, Van Marck E, Huget P, et al Arterio-venous gradients of IL-6, plasma and serum VEGF and D-dimers in human cancer. British journal of cancer. 2002;87(12):1437–44. 10.1038/sj.bjc.6600655 12454774 PMC2376277

[34] Hastings JM, Licence DR, Burton GJ, Charnock-Jones DS, Smith SK. Soluble vascular endothelial growth factor receptor 1 inhibits edema and epithelial proliferation induced by 17beta-estradiol in the mouse uterus. Endocrinology. 2003;144(1):326–34. 10.1210/en.2002-220641 . 12488361

[35] Uda Y, Hirano T, Son G, Iimuro Y, Uyama N, Yamanaka J, et al Angiogenesis is crucial for liver regeneration after partial hepatectomy. Surgery. 2013;153(1):70–7. 10.1016/j.surg.2012.06.021 . 22862899

[36] Hanahan D, Folkman J. Patterns and emerging mechanisms of the angiogenic switch during tumorigenesis. Cell. 1996;86(3):353–64. . 8756718 10.1016/s0092-8674(00)80108-7

[37] Starlinger P, Assinger A, Haegele S, Wanek D, Zikeli S, Schauer D, et al Evidence for serotonin as a relevant inducer of liver regeneration after liver resection in humans. Hepatology. 2014;60(1):257–66. 10.1002/hep.26950 . 24277679

[38] Starlinger P, Haegele S, Offensperger F, Oehlberger L, Pereyra D, Kral JB, et al The Profile of Platelet alpha-Granule Released Molecules Affects Postoperative Liver Regeneration. Hepatology. 2015 10.1002/hep.28331 . 26528955

[39] Matsuo R, Nakano Y, Ohkohchi N. Platelet administration via the portal vein promotes liver regeneration in rats after 70% hepatectomy. Annals of surgery. 2011;253(4):759–63. . 21475016 10.1097/SLA.0b013e318211caf8

[40] Karayiannakis AJ, Syrigos KN, Polychronidis A, Zbar A, Kouraklis G, Simopoulos C, et al Circulating VEGF levels in the serum of gastric cancer patients: correlation with pathological variables, patient survival, and tumor surgery. Annals of surgery. 2002;236(1):37–42. 12131083 10.1097/00000658-200207000-00007 PMC1422546

[41] Ding BS, Nolan DJ, Butler JM, James D, Babazadeh AO, Rosenwaks Z, et al Inductive angiocrine signals from sinusoidal endothelium are required for liver regeneration. Nature. 2010;468(7321):310–5. 10.1038/nature09493 21068842 PMC3058628

[42] LeCouter J, Moritz DR, Li B, Phillips GL, Liang XH, Gerber HP, et al Angiogenesis-independent endothelial protection of liver: role of VEGFR-1. Science. 2003;299(5608):890–3. 10.1126/science.1079562 . 12574630

[43] DeLeve LD. Liver sinusoidal endothelial cells and liver regeneration. The Journal of clinical investigation. 2013;123(5):1861–6. 10.1172/JCI66025 23635783 PMC3635729

