# Supplementary figures and images for: A Switch in the Dynamics of Intra-Platelet VEGF-A from Cancer to the Later Phase of Liver Regeneration after Partial Hepatectomy in Humans
Source: PLoS One. 2016 Mar 1;11(3):e0150446. doi: 10.1371/journal.pone.0150446 (PMC4773068; doi:10.1371/journal.pone.0150446)

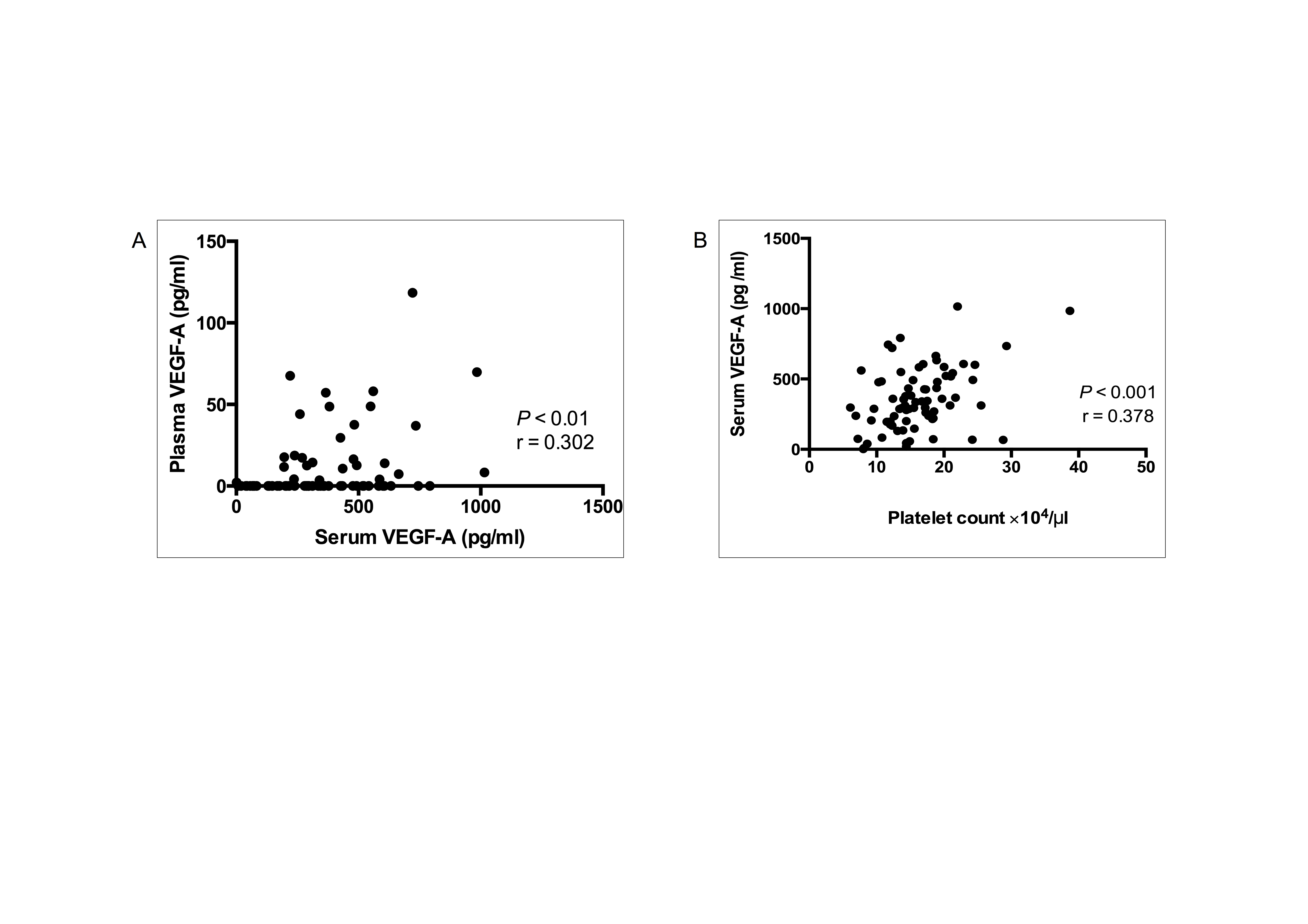

Supplement: S1 Fig — Correlation between serum and plasma VEGF-A (A). Correlation between platelet count and serum VEGF-A (B). (Variables included from both preoperative and postoperative events). (TIFF) [file pone.0150446.s001.tiff]

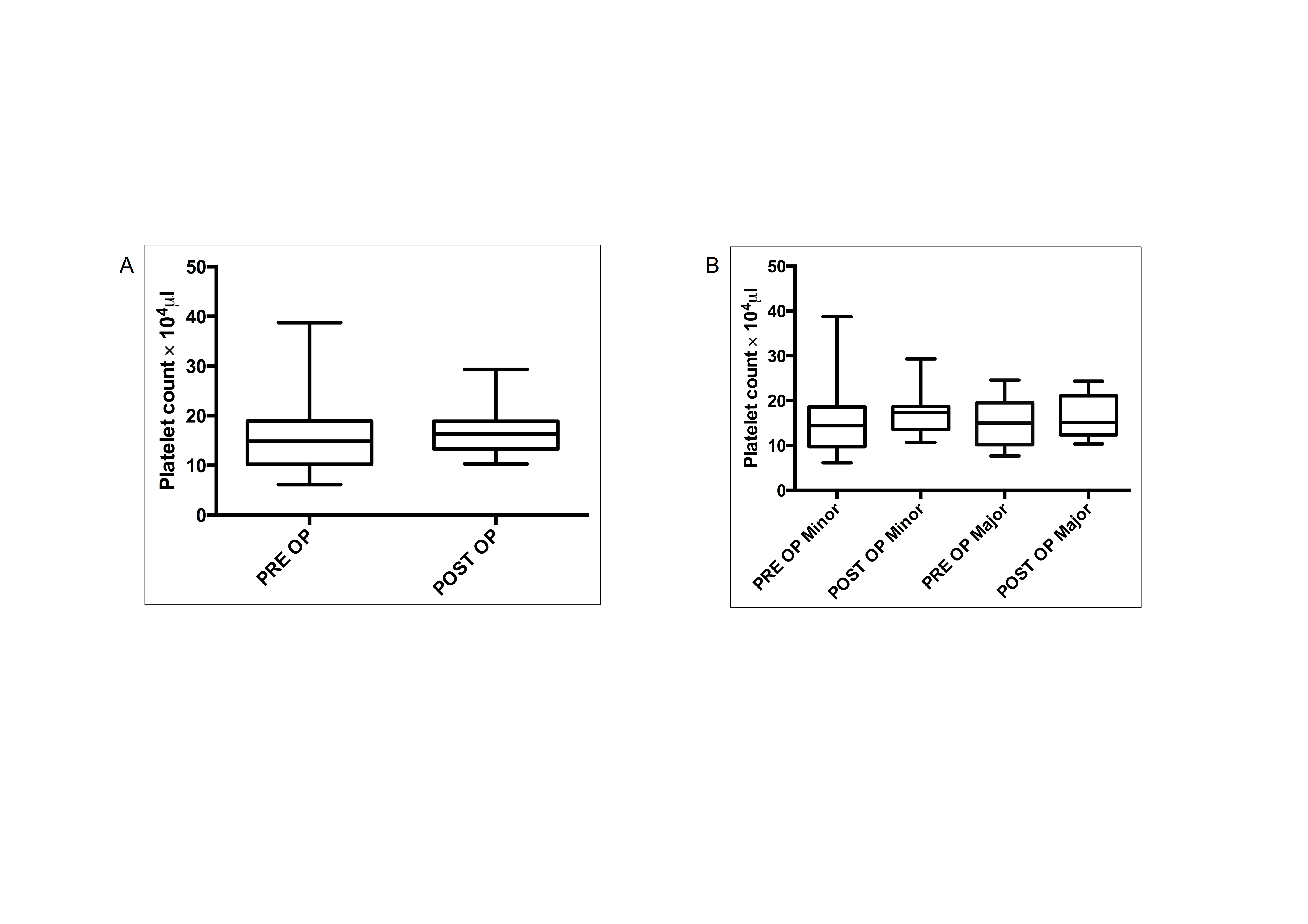

Supplement: S2 Fig — Platelet count before (PRE OP) and four weeks after operation (POST OP) (A). Platelet count in major and minor groups, before and four weeks after operation (B). (TIFF) [file pone.0150446.s002.tiff]

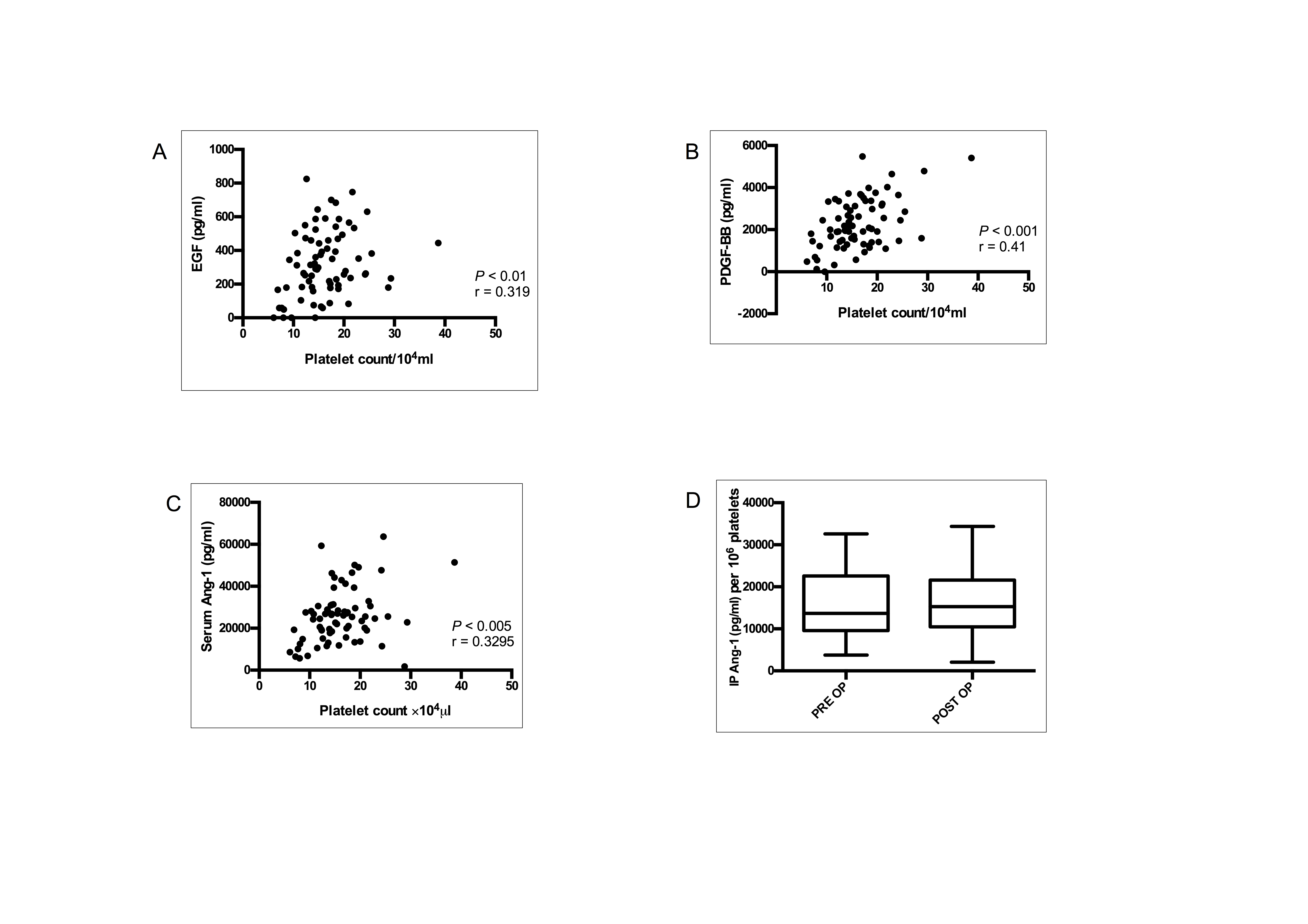

Supplement: S3 Fig — Correlation between platelet count and serum EGF (A), PDGF-BB (B), Ang-1 (C). IP Ang-1 concentrations were analyzed preoperatively (PRE OP) and postoperatively (POST OP) (D). IP Ang-1 was expressed per 106 platelets. (TIFF) [file pone.0150446.s003.tiff]

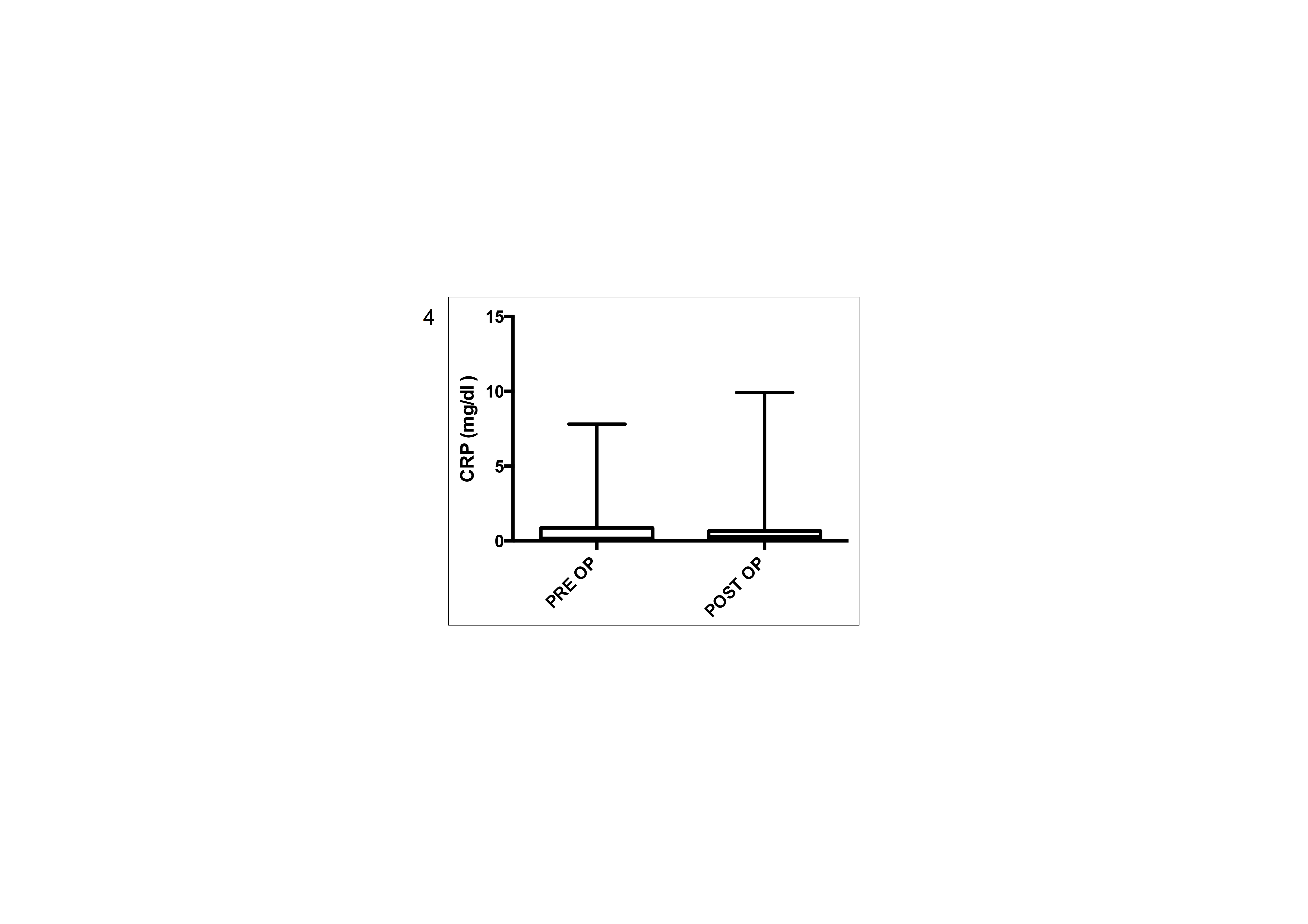

Supplement: S4 Fig — CRP level before (PRE OP) and four weeks after operation (POST OP) (N = 28). (TIFF) [file pone.0150446.s004.tiff]
